# Serotypes, Antibiotic Susceptibility, Genotypic Virulence Profiles and SpaA Variants of *Erysipelothrix rhusiopathiae* Strains Isolated from Pigs in Poland

**DOI:** 10.3390/pathogens12030409

**Published:** 2023-03-03

**Authors:** Marta Dec, Dominik Łagowski, Tomasz Nowak, Dorota Pietras-Ożga, Klaudia Herman

**Affiliations:** 1 Department of Veterinary Prevention and Avian Diseases, Faculty of Veterinary Medicine, University of Life Sciences in Lublin, 20-033 Lublin, Poland; 2 Department of Veterinary Microbiology, Faculty of Veterinary Medicine, University of Life Sciences in Lublin, 20-033 Lublin, Poland; 3 Diagnostic Veterinary Laboratory Vet-Lab Brudzew Dr. Piotr Kwieciński, Department of Molecular Biology, 62-720 Brudzew, Poland; 4 Department of Epizootiology and Clinic of Infectious Diseases, Faculty of Veterinary Medicine, University of Life Sciences in Lublin, 20-612 Lublin, Poland

**Keywords:** erysipelas, pigs, antibiotic susceptibility, serotype, virulence genes, vaccine strain

## Abstract

The aim of the study was phenotypic and genotypic characterization of *Erysipelothrix rhusiopathiae* strains isolated from diseased pigs in Poland and comparison of the SpaA (Surface protective antigen A) sequence of wild-type strains with the sequence of the R32E11 vaccine strain. The antibiotic susceptibility of the isolates was assessed using the broth microdilution method. Resistance genes, virulence genes, and serotype determinants were detected using PCR. The *gyrA* and *spaA* amplicons were sequenced to determine nonsynonymous mutations. The *E. rhusiopathiae* isolates (n = 14) represented serotypes 1b (42.8%), 2 (21.4%), 5 (14.3%), 6 (7.1%), 8 (7.1%), and N (7.1%). All strains were susceptible to β-lactams, macrolides and florfenicol. One isolate showed resistance to lincosamides and tiamulin, and most strains were resistant to tetracycline and enrofloxacin. High MIC values of gentamicin, kanamycin, neomycin, trimethoprim, trimethoprim/sulfadiazine, and rifampicin were recorded for all isolates. Phenotypic resistance was correlated with the presence of the *tetM*, *int-Tn*, *lasE*, and *lnuB* genes. Resistance to enrofloxacin was due to a mutation in the *gyrA* gene. All strains contained the *spaA* gene and several other genes putatively involved in pathogenesis (*nanH.1*, *nanH.2*, *intl*, *sub*, *hlyA*, *fbpA*, ERH_1356, *cpsA*, *algI*, *rspA* and *rspB*) Seven variants of the SpaA protein were found in the tested strains, and a relationship between the structure of SpaA and the serotype was noted. *E. rhusiopathiae* strains occurring in pigs in Poland are diverse in terms of serotype and SpaA variant and differ antigenically from the R32E11 vaccine strain. Beta-lactam antibiotics, macrolides, or phenicols should be the first choice for treatment of swine erysipelas in Poland. However, due to the small number of tested strains, this conclusion should be approached with caution.

## 1. Introduction

*Erysipelothrix rhusiopathiae* is the aetiological agent of erysipelas, one of the most well-known infectious diseases in pigs. The pathogen can also infect poultry and other groups of animals, as well as humans. Despite advanced veterinary care, cases of porcine erysipelas still occur on farms worldwide, causing significant economic losses [1]. The disease affects both growing pigs over 3 months of age and adult pigs. It can be acute, subacute, or chronic, but subclinical infections without signs of disease occur as well. In the acute course of the disease, sepsis and sudden unexpected deaths occur in the herd. Other pigs may have high fever, depression, and mobility problems. The subacute form is also septicaemic but is clinically less severe than the acute form. The characteristic symptoms are pink, red, or purple skin lesions (‘diamond skin’), which may disappear within a few days. The chronic forms of porcine erysipelas may follow acute outbreaks or develop insidiously. It is generally manifested by enlarged, painful joints, lameness, or valvular endocarditis. Pigs with valvular lesions may show few clinical signs, that may include respiratory failure, lethargy, and cyanosis during exercise [1,2]. In humans, infection with *E. rhusiopathiae* most commonly takes the form of a skin condition known as erysipeloid. Pig farmers, veterinarians, and slaughterhouse workers are among the most at-risk groups [3,4]. In Poland, in the period from 1999 to 2008, when the registration of *E. rhusiopathiae* infections in humans was obligatory, 1121 cases were recorded (0.25/100,000) [5]. 

*E. rhusiopathiae* is a facultative anaerobic, non-spore-forming, slender, gram-positive rod that can survive within macrophages [4,6]. It belongs to the genus *Erysipelothrix*, that currently includes 10 species (*E. rhusiopathiae*, *E. tonsillarum*, *E. inopinata*, *E. piscisicarius* sp. Nov, *Erysipelothrix* sp. Strain 1, *Erysipelothrix* sp. Strain 3, *E. larvae*, *E. anatis*, *E. urinaevulpis*, and *E. aquatica*) [7,8]. The genome of *E. rhusiopathiae* (1,787,941 bp) is one of the smallest within the phylum *Firmicutes* [9]. Swine are the most important reservoir of *E. rhusiopathiae*. It has been estimated that 10.5% to even 98% of asymptomatic pigs carry *E. rhusiopathiae* in their tonsils [10,11,12,13]. Healthy carriers and recovered and chronically infected pigs may shed the pathogen in their faeces or oronasal secretions [3]. Under laboratory conditions, *E. rhusiopathiae* grows on enriched media supplemented with blood, serum, or 0.1% Tween 80, in the form of small, greyish translucent colonies. It favours an alkaline pH from 7.2 to 7.6 and a temperature of 30–37 °C [2,4]. Based on peptidoglycan antigens, *E. rhusiopathiae* strains are classified into 17 serotypes, i.e., 1a, 1b, 2, 4, 5, 6, 8, 9, 11, 12, 15, 16, 17, 19, 21, 23 and type N, which does not produce precipitating antibodies in rabbits [7]. 

The precise mechanism by which *E. rhusiopathiae* causes disease is speculative, but several potential virulence factors have been identified, including neuraminidase, capsular antigen, rhusiopathiae surface protein A (RspA) and B (RspB), haemolysin, hyaluronidase, surface protective antigen (Spa), phospholipase, internalin-like protein HP1472, and some others [1,14,15,16]. It is likely that neuraminidase, an enzyme that cleaves sialic acid from sialo-glyco-conjugates, mediates the widespread vascular damage that accompanies swine erysipelas. Vascular damage leads to thrombosis and interference with microcirculation in capillaries and venules at many sites [14].

Spa is regarded as a key immunogen, and anti-Spa antibodies are known to play an important role in protecting the host against erysipelas. Spa shows structural diversity and occurs in three types, SpaA, SpaB, and SpaC, of which SpaA is the most frequently identified. Immunization with a particular Spa type has been shown to provide protection against disease development following exposure to strains containing homologous Spa, whereas protection against infection with strains carrying another Spa type is limited [17,18]. Therefore, determination of the type of Spa protein in wild-type strains is of epidemiological importance. Several SpaA variants have been distinguished based on nonsynonymous mutations found in the *spaA* region, corresponding to the immunoprotective domain, and the virulence of *E. rhusiopathiae* strains may be dependent on the SpaA variant [19]. SpaA promotes the adherence of *E. rhusiopathiae* to the endothelial cells and increases resistance to phagocytosis [20,21,22]. 

Antibiotic therapy and vaccinations play a decisive role in the control of infections with *E. rhusiopathiae* strains in pigs. Penicillin is the drug of choice in the treatment of porcine erysipelas. Commercial vaccines contain inactivated bacteria (bacterins), most commonly serotype 2, and are believed to provide high protection against serotype 2 and 1 strains, which cause most clinical cases in swine [3]. Several inactivated swine erysipelas vaccines are available in Poland (Table S1), of which the most popular include ERYSENG^®^ and ERYSENG^®^ PARVO (Laboratorios Hipra S.A., Amer, Spain), both of which contain the *E. rhusiopathiae* R32E11 strain.

The aim of the study was to determine the serotypes, antibiotic susceptibility, and genotypic virulence profiles of *E. rhusiopathiae* isolates from pigs and to compare wild strains and the R32E11 vaccine strain in terms of the structure of the SpaA immunogen. Effective infection control requires knowledge of the characteristics of the etiological agent, and currently there are no reports of *E. rhusiopathiae* strains from pigs in Poland. During disease outbreaks, correctly identifying phenotypic and genetic differences between isolates is critical to better understanding disease epidemiology.

## 2. Materials and Methods

### 2.1. Isolation of Erysipelothrix rhusiopathiae Strains

*E. rhusiopathiae* strains were isolated post-mortem from tissues from 14 pigs bred on various farms located in three provinces in Poland between 2017 and 2022. The age of the animals ranged from 4 to 10 months (Table S2). Nine pigs from small backyard farms (No. 1, 3, 4, 6, 7, 10, 11, 12, 13, and 14, corresponding to *E. rhusiopathiae* strains 1S, 3S, 4S, 6S, 7S, 10S, 11S, 12S, 13S, and 14S, respectively) were not vaccinated against erysipelas, while 4 pigs from large pig farms (No. 2, 5, 8, and 9, corresponding to strains 2S, 5S, 8S, and 9S, respectively) were vaccinated (information obtained from veterinarians or breeders shows that the ERYSENG^®^ PARVO vaccine, Hipra, was used most often, but precise information in this regard is not known). Most pigs showed no obvious signs of disease. However, some animals admitted to the slaughterhouse developed skin lesions, probably due to post-transport stress (Figure S1). The veterinary history showed that several pigs had previously suffered from erysipelas and been treated with antibiotics. Samples were taken at the slaughterhouse from pork half-carcasses. The post-mortem examination revealed skin lesions or thickening in the subcutaneous tissue (Table S2). Carcasses of all individuals with symptoms of erysipelas were disposed of. Bacteria were isolated from nodular lesions located deep in the subcutaneous tissue, pathologically altered heart valves, muscular fascia, and lymph nodes located closest to the affected sites or synovial fluid. The collected tissues were homogenized in glass mortars and then suspended in TKT Edwards modified broth with 5% haemolysed sheep blood (Biomaxima, Lublin, Poland). After 48 h incubation at 37 °C, the material was plated on blood agar (GRASO Biotech, Owidz, Poland) and incubated at 37 °C for 48 h. Cultures growing as small, greyish translucent colonies and having the morphology of slender gram-positive rods were considered *E. rhusiopathiae*. Alpha haemolysis was observed for most strains (Figure S1). Pure cultures were propagated on BHI broth with the addition of 0.1% Tween 80 (Merck, Warsaw, Poland). 

### 2.2. Identification of E. rhusiopathiae Strains

The collected isolates grown on Columbia agar supplemented with 5% blood (BTL, Łódź, Poland) were identified by matrix-assisted laser desorption ionization-time of flight (MALDI-TOF) mass spectrometry (MS) using a standard ethanol/formic acid extraction method [23]. The mass spectra obtained from each isolate were processed with the MALDI Biotyper^®^ 3.1 database (Bruker Daltonics, Bremen, Germany), which contains 8468 mass spectra of reference strains, including 10 strains of *E. rhusiopathiae*. Identification to species level was considered reliable with log(score) ≥ 2.000 [23].

### 2.3. Serotyping of E. rhusiopathiae Strains

Serotyping was performed based on four multiplex PCR protocols [7,24]. The sequence of primers, annealing temperature, and size of PCR products are listed in Table S3. PCR reactions were performed using DreamTaq Green DNA Polymerase (Thermo Fisher Scientific Baltics UAB, Vilnius, Lithuania). Reference *E. rhusiopathiae* strains (Fujisawa serotype 1a, ATCC 19414 serotype 2, R32E11 serotype 2, Tuzok serotype 6, and Bano serotype 21) were used as positive controls. Strain ATCC 19414 was obtained from Argenta (Poznań, Poland) and other reference strains were provided in the form of genomic DNA by Dr. Shimoji, National Institute of Animal Health, Japan.

### 2.4. Antimicrobial Susceptibility Testing

The antibiotic susceptibility of isolates was determined by the broth microdilution procedure using 17 antimicrobial agents: ampicillin, ceftiofur, tetracycline, erythromycin, tylosin, clindamycin, lincomycin, tiamulin, enrofloxacin, streptomycin, spectinomycin, gentamicin, kanamycin, neomycin, trimethoprim, trimethoprim and sulfadiazine (1:5), and rifampicin. All antimicrobial agent powders were obtained from Merck (Warsaw, Poland). Ready-to-use solutions of tiamulin (Biomutin, 200 mg/mL) and spectinomycin (100 mg/mL) were purchased from BIOWET DRWALEW S.A. (Drwalew, Poland) and Merck (Warsaw, Poland), respectively.

Inocula were prepared by suspending bacteria in 0.85% NaCl to obtain a density of 0.5 McFarland. A 150 µL volume of the inoculum was added to 10 mL of BHI broth (BTL, Łódź, Poland) containing 0.1% Tween 80. Microdilution plates were inoculated with 50 μL of bacterial suspension and 50 μL of the appropriate antibiotic concentration. Plates were incubated at 36 °C in 5% CO_2_ for 45 h, and MIC values were read as the lowest concentration of an antimicrobial agent at which visible growth was inhibited. The reference strain ATCC 19414 was analysed in parallel with the wild strains. The quality control of antimicrobial substances was carried out using *E. coli* strain ATCC 25922 and Müller–Hinton broth [25].

Categorization of *E. rhusiopathiae* strains as susceptible, intermediately resistant, and resistant was carried out based on CLSI guidelines (document Vet06, 2017) [26]. For some antimicrobials not included in this guide, breakpoints recommended for other antibiotics of the same class or for other types of gram-positive bacteria have been adopted (Table 1). In the case of aminoglycoside antibiotics (gentamicin, kanamycin, neomycin, streptomycin, and spectinomycin), folic acid inhibitors (trimethoprim and trimethoprim/sulfadiazine), and rifampicin, no cut-off points were proposed due to high MIC values or/and their unimodal distribution. 

### 2.5. Isolation of DNA

Whole-genome DNA was extracted from *E. rhusiopathiae* strains using the Gene MATRIX Bacterial and Yeast Genomic DNA Purification Kit (Eurx, Gdańsk, Poland) following the manufacturer’s protocol, modified to extend the incubation time of bacteria in the lysis buffer from 15 min to 1 h (gram-positive bacteria, unlike gram-negative bacteria, have a thick cell wall, the removal of which requires a longer incubation of the bacteria with lytic enzymes). DNA concentration was determined using the NanoDrop Lite spectrophotometer (Thermo Fisher Scientific, Waltham, MA, USA), and the quality of DNA was checked by agarose (1.5% *w*/*v*) gel electrophoresis. The final DNA concentration was ~18 ng/µL. 

### 2.6. Detection of Resistance Genes

PCR (singleplex or multiplex) based on gene-specific primers was used to detect the presence of 19 genes that confer resistance to aminoglycosides (*aac(6′)-Ie-aph(2″)-Ia*, *aph(3′)-IIIa*, *ant(4′)-Ia*, *aph(2″)-Ib*, *aph(2″)-Ic*, *aph(2″)-Id*, *ant(6)-Ia*, *ant(9)-Ia*, and *aadK*), tetracycline (*tetK*, *tetL*, *tetM*, and *tetO*), macrolides and lincosamides (*ermA*, *ermB*, *mefA/E*, and *lnuB*), pleurumutilins (*lsaE*), chinolones (*gyrA*), and the Tn916/Tn1 integrase gene (*int-Tn*) (Table S3). The primers for amplification of the *gyrA* and *aadK* genes were designed using the Primer-BLAST tool (https://www.ncbi.nlm.nih.gov/tools/primer-blast/, accessed on 4 November 2022). DNA amplification was performed using DreamTaq DNA polymerase (Thermo Fisher Scientific, Vilnius, Lithuania) under the following conditions: 5 min at 95 °C, 30 cycles of 50 s at 95 °C, 40 s at 50–60 °C (according to the annealing temperature for the individual sets of primers as listed in Table S4), 60 s/1000 bp at 72 °C, and 8 min of final extension at 72 °C. The composition of the reaction mixture was selected based on the recommendations of the polymerase manufacturer. Wild strains of *E. rhusiopathiae* and lactic acid bacteria containing resistance genes were used as a positive control (Table S5). 

### 2.7. Sequence Analysis of the gyrA Gene

The sequencing of the PCR products (613 bp fragment of the *gyrA* gene) of representative enrofloxacin-susceptible and enrofloxacin-resistant strains was carried out using the Sanger method in the external service laboratory of Nexbio Sp. z o.o. (Lublin, Poland). Chromatograms were analysed using Chromas Lite (ver. 2.6.6, Technelysium Pty Ltd., South Brisbane, Australia), and DNA sequences of *gyrA* genes were deposited in GenBank (Accession Nos. OP921301–OP921305). Amino acid (aa) sequences were predicted using the NCBI translate tool ORF finder (https://www.ncbi.nlm.nih.gov/orffinder/, accessed on 26 November 2022). The ClustalW Multiple Alignment tool (MEGA X software, https://www.megasoftware.net/, accessed on 26 November 2022) was used to align predicted aa sequences. The *gyrA* sequence of reference strain *E. rhusiopathiae* ATCC 19414 (enrofloxacin-susceptible) was retrieved from the NCBI GenBank database (Acc. No. LR134439.1).

### 2.8. Detection of Virulence Genes

Singleplex or multiplex PCR, using gene-specific primers, was used to detect the presence of 14 genes associated with *E. rhusiopathiae* virulence traits (*spaA*, *spaB*, *spaC*, *nanH.1*, *nanH.2*, *cpsA*, ERH_1356, *intl-like*, *rspA*, *rspB*, *algI*, *sub*, *hlyA*, *fbpA*, and *hlyIII*). The genes were selected based on previous reports of their confirmed or putative role in the pathogenesis of *E. rhusiopathiae* infections [1,15,16,29,30]. The primers that were used for the first time in this study were designed using the Primer-BLAST tool (https://www.ncbi.nlm.nih.gov/tools/primer-blast/, accessed on 11 December 2022). Primer sequences, PCR product size, and annealing temperature are shown in Table S6.

DNA amplification was performed using DreamTaq Green DNA Polymerase (Thermo Fisher Scientific Baltics UAB, Vilnius, Lithuania) and the T100 thermal cycler (Bio-Rad, Hercules, CA, USA) under the following conditions: 5 min at 94 °C, 30 cycles of 45 s at 95 °C, 40 s at 50-56 °C (Table S6), 60 s/1000 bp at 72 °C, and 8 min of final extension at 72 °C. PCR products were separated by electrophoresis in 1.5% agarose and their profiles were analysed with Image Lab Software (BioRad, Hercules, CA, USA) by comparison with the Nova 100 bp DNA ladder (Novazym, Poznań, Poland). Reference strains of *E. rhusiopathiae* Fujisawa (*spaA*+, *nanH.1*+, *nanH.2+*, *cspA+*, ERH_1356+, ERH_1472+, *rspA+*, *rspB+*, *algI+*, *hlyA+*, *hlyIII+*, *fbpA+*, and *sub+*) (GenBank Acc. No. AP012027.1), Tuzok (*spaB*+) and *E. piscisicarius* 715 (*spaC*+) were used as a positive control. 

### 2.9. Whole spaA gene Amplification and Sequence Analysis

Amplification of the entire *spaA* gene was performed using primers Spa-fw (5′-ATGAAAAAGAAAAAACACCTA-3′) and Spa-rv (5′-CTATTTTAAACTTCCATCGTT-3′) [16] and DreamTaq polymerase (Thermo Fisher Scientific Baltics UAB, Vilnius, Lithuania) at an annealing temperature of 49 °C. In addition to the wild-type strains (n=14), the R32E11 strain, that is used in the production of the ERYSENG^®^ and ERYSENG^®^ PARVO vaccines (Laboratorios Hipra S.A., Amer, Spain), was also used in the study. DNA of the R32E11 strain was provided by Dr. Shimoji, National Institute of Animal Health, Japan. PCR products were sequenced using the Sanger method in the external service laboratory of Nexbio Sp. z o.o. (Lublin, Poland). The DNA SequenceReverse and Complement Online Tool (http://www.cellbiol.com, accessed on 24 November 2022) was used to determine the consensus sequence of the *spaA* gene, and aa sequences were predicted using the NCBI translate tool ORF finder (https://www.ncbi.nlm.nih.gov/orffinder/, accessed on 26 November 2022). DNA sequences of *spaA* genes were deposited in GenBank (Accession Nos. OP822679-OP822691, OQ054982 and MZ448116). 

The ClustalW Multiple Alignment tool (MEGA X software) was used to align the predicted aa sequences of the SpaA of different isolates. To determine the evolutionary distance between the strains, a comparative analysis of the 447-aa N-terminal fragment (covering 29 aa-signal sequence + 384 aa-immunoprotective domain + 34 aa-proline-rich domain) of the SpaA protein sequence was performed. The *spaA* sequences of reference *E. rhusiopathiae* strains and several wild-type strains isolated from pigs (in various countries) and water poultry (in Poland) included in the analysis (n = 17) were retrieved from the NCBI GenBank database (Table S7). Clustering was conducted in MEGA X using the maximum likelihood method with a bootstrap support value of 500. All positions containing missing data were eliminated (complete deletion option). Due to the cut-off of illegible initial segments of the chromatograms, there were a total of 435 positions in the final dataset (the first 12 amino acids of the SpaA corresponding to the signal domain were missing). 

## 3. Results

### 3.1. Identification of E. rhusiopathiae Isolates

The bacteria isolated from porcine tissues were identified by mass spectrometry as *E. rhusiopathiae* (n = 14), and the log(score) for all samples was in the range of 2.028 to 2.312.

### 3.2. Serotyping

**Figure 1:**
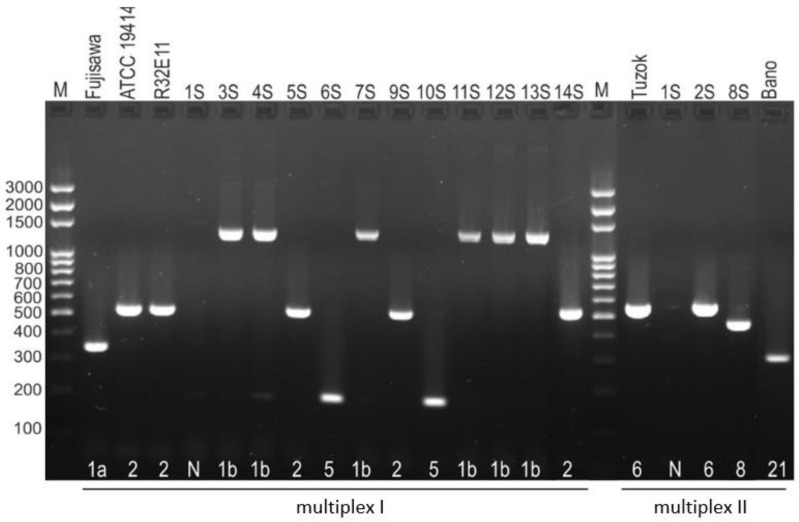
Serotypic differentiation of *E. rhusiopathiae* strains by multiplex PCR. The strains ATCC 19414 serotype 2, R32E11 serotype 2, Tuzok serotype 6, and Bano serotype 21 were used as positive control; M—molecular size marker.

In the serotyping test, positive PCR reactions were obtained for 13 of 14 strains of *E. rhusiopathiae*. Nearly half of the tested isolates belonged to serotype 1b (6/14; 42.8%). The remaining isolates represented serotype 2 (3/14; 21.4%), serotype 5 (2/14; 14.3%), serotype 6 (1/14; 7.1%), and serotype 8 (1/14; 7.1%). One isolate for which no PCR product was obtained in any of the four multiplex PCR reactions was designated serotype N (1/14; 7.1%) (Figure 1).

### 3.3. Antibiotic Susceptibility

All strains were susceptible to ampicillin, ceftiofur, erythromycin, and tylosin. One isolate (8S) (1/14; 7.1%) was resistant to clindamycin, lincomycin, and tiamulin, and most strains showed resistance to tetracycline (10/14, 71.4%) and enrofloxacin (57.1%). Interestingly, all strains susceptible to tetracycline (n = 4) were also susceptible to enrofloxacin (Table 2).

High minimum inhibitory concentrations (>512 µg/mL) of gentamicin, kanamycin, neomycin, trimethoprim, trimethoprim/sulfadiazine, and rifampicin (>128 µg/mL) were obtained for all isolates. This result, combined with unimodal distribution of MICs, indicates that *E. rhusiopathiae* bacteria are naturally resistant to these antimicrobials. The MIC values of streptomycin and spectinomycin ranged from 32 to 256 and 16 to 128 µg/mL, respectively (Table 2). 

### 3.4. Genotypic Resistance Profiles

Genotypic resistance profiles were compatible with phenotypic resistance. All tetracycline-resistant isolates contained the *tetM* gene (coding for ribosomal protection protein, which catalyses the release of tetracycline from ribosomes in a reaction dependent on GTP) and the Tn916/Tn1 transposon integrase gene. The 8S strain resistant to lincosamides and tiamulin contained genes *lnuB* (coding for lincosamide nucleotidyltransferase) and *lsaE* (coding for ABC-F efflux pump) (Table 2). The *aadK* gene encoding aminoglycoside nucleotidyltransferase was detected in all isolates; however, based on the results (unimodal distribution of MIC values), its role in the resistance of *E. rhusiopathiae* strains to aminoglycoside antibiotics remains unclear. None of the seven other considered genes determining resistance to aminoglycosides were detected in the isolates tested (Table 2). Sequence analysis of the *gyrA* gene showed that resistance of *E. rhusiopathiae* strains to enrofloxacin (MICs 8–16 µg/µL) is due to a mutation at position 257 (ACA→ATA or ACA→AAA), which translates into a change in the aa sequence, Thr86→Ile or Thr86→Lys86 (Table 3).

### 3.5. Virulence-Associated Genes

The genotypic virulence profiles of the tested strains of *E. rhusiopathiae* were identical. All isolates contained the *spaA* gene encoding the immunogenic SpaA protein, the *nanH.1* and *nanH.2* neuraminidase genes, and other genes whose products may enable invasion of host tissues (*intl*, *sub*, *hlyA*, *fbpA*, ERH_1356, *rspA*, and *rspB*) or determine resistance to attack by complement and phagocytic cells (*cpsA* and *algI*) (Table 4).

### 3.6. SpaA Sequence Analysis

The size of the *spaA* amplicon in the tested *E. rhusiopathiae* isolates ranged from 1761 to 1881 bp (corresponding to 587–626 aa), while in the case of the reference R32E11 strain it was ~2200 bp. This variation in the size of the PCR products was noticeable in the electropherogram (data not shown) and resulted from the different number of 60-nucleotide tandem repeats of the DNA sequence at the 3′ end of the *spaA* gene.

In most of the tested strains (9/14; 64.3%, serotypes 1b, 2, 5, 6, and N), there were nine 20-aa repeats containing a GW module (corresponding to a *spaA* gene length of 1881 nt) in the predicted SpaA sequence, as in the reference Fujisawa and ATCC 19414 strains. Four strains (4/14; 28.6%) representing serotype 1b (4S, 11S, 12S, and 13S) contained 8 tandems (corresponding to a total *spaA* gene length of 1818 nt) and one strain (1/14; 7.1%) (8S, serotype 8) contained 7 tandems (total *spaA* gene length 1761 nt).

Signal sequences spanning 29 aa were conserved among the SpaA protein of all isolates. However, it should be noted that the SpaA sequences obtained in this work were missing the first 11 or 12 aa (hence the analysed signal sequences contained only 17 aa) due to the need to remove the illegible ends of the chromatograms.

Following a comparative analysis of wild-type *E. rhusiopathiae* strains and R32E11 vaccine strain, as well as the highly virulent Fujisawa strain [9] and the reference strain ATCC 19414, 15 nonsynonymous mutations were found within the SpaA hypervariable domain (384-aa in length; region 30–413 aa) and the proline-rich region (34-aa in length; 414–447 aa). On this basis, 7 variants of the SpaA protein were distinguished in the wild-type strains (Table 5). 

SpaA hypervariable region sequences (immunoprotective domain, SpaA antigen) of all wild-type *E. rhusiopathiae* strains differed from those of the R32E11 vaccine strain. The difference concerned one (strain 14S), two (1S), six (2S), seven (3S, 4S, 5S, 6S, 7S, 9S, 10S, 11S, and 13S), eight (8S) or even nine (12S) aa; taking into account the proline-rich domain as well, the difference increases to 8 aa in the case of the 5S and 9S strains. All strains representing serotypes 1b and 5, except the 12S strain, had an identical aa sequence in the SpaA immunoprotective domain. The variation at position 38 (Pro38→Gln) was unique to the strain 1S serotype N, the variation at position 54 (Gly54→Ala) was found only in the strain 8S serotype 8, polymorphism at positions 139 (Gln139→Lys) and 232 (Ile232→Thr) reported only in the 12S strain, and the variation at position 423 (Pro423→Gln) was unique to the 5S and 9S strains of serotype 2 (Table 5).

As with the R32E11 vaccine strain, none of the wild-type isolates showed 100% homology to the Fujisawa strain in terms of immunoprotective domain and proline-rich region sequences. The highest homology was found in the 2S serotype 6 strain, in which variation was noted in only three aa positions (257, 426, 435). The remaining isolates differed by 4–6 aa from the Fujisawa strain (Table 5). 

The sequence of the 14S strain was identical to that of the reference strain ATCC 19414 (isolated from the spleen of a pig with endocarditis in 1950), and the 1S strain serotype N differed from the ATCC 19414 strain in only one aa (Gln38) (Table 5).

**Figure 2:**
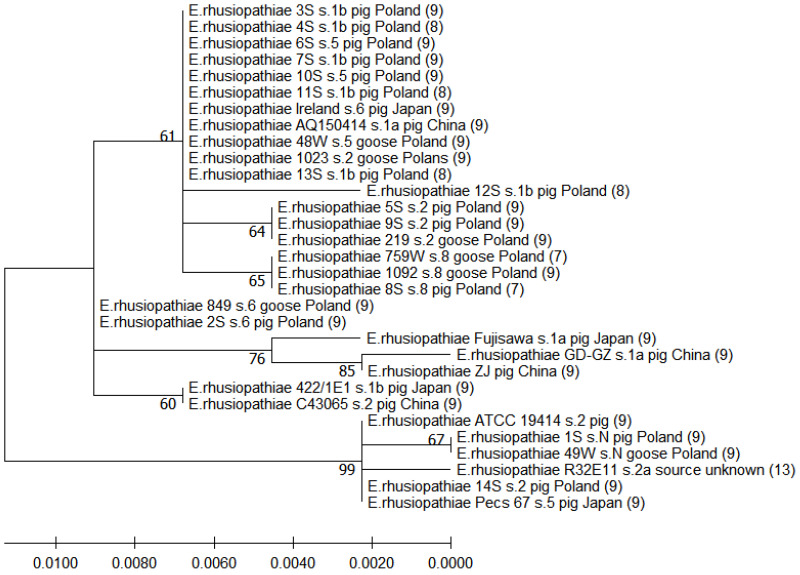
Dendrogram showing the similarity among the predicted aa sequences of signal, immunoprotective, and proline-rich domains of SpaA built by the maximum likelihood method. The percentage of replicate trees in which the associated taxa were clustered together in the bootstrap test (500 replicates) is shown next to the branches. Scale bars show genetic distance. Numbers in parentheses indicate the number of repetitive tandems in the C-terminal region of SpaA (these data were taken from another analysis involving alignment of complete SpaA sequences).

Comparative analysis of 31 strains of *E. rhusiopathiae* (14 strains tested in this study and 17 additional strains) showed that the sequence of the N-terminal 447-aa region of SpaA (including signal, immunoprotective, and proline-rich domains) in 7 of the 14 tested strains, representing serotype 1b or 5, was homologous with the sequence of *E. rhusiopathiae* strains isolated from pigs in China (AQ 150414 serotype 1a) and Japan (Ireland serotype 6), as well as from geese in Poland (1023 serotype 2, 48W serotype 5). The clustering revealed a relationship between the serotype and the sequence of the immunoprotective domain of the SpaA protein. The strain 8S serotype 8 clustered with the two *E. rhusiopathiae* goose strains (759W and 1092) representing serotype 8, while the 2S serotype 6 strain had an identical aa sequence to that of strain 846 serotype 6 isolated from geese in Poland. The two serotype 2 strains (5S and 9S) formed a common cluster with the goose serotype 2 strain 219. On the other hand, two other serotype 2 strains, 14S and 1023, formed clades with strains of different serotypes. The sequence of the strain 1S serotype N was homologous with the sequence of strain 49W serotype N isolated from domestic goose in Poland. The dendrogram also shows that, among the total 31 analysed strains representing 7 serotypes (1a, 1b, 2, 5, 6, 8, and N), 7 and 8 tandem repeats in the C-terminal region of SpaA were present only in strains of serotype 8 (2/3) and 1b (4/6), respectively (Figure 2).

## 4. Discussion

### 4.1. Occurrence of E. rhusiopathiae Infections in Pigs

Most of the cases of erysipelas reported in this study were detected in unvaccinated pigs from backyard farms. The animals apparently showed no symptoms, or only mild symptoms, of the disease, and the infection was found only during the post-mortem examination. Some of the pigs included in the study had previously been treated for symptoms suggestive of erysipelas. It is therefore possible that antibiotic therapy did not have a completely curative effect in these individuals, and the disease became chronic. Vaccination against swine erysipelas in Poland is not mandatory and is usually not performed on backyard farms for economic reasons. The cost of the smallest package of vaccine can be higher than the cost of antibiotic therapy. The occurrence of erysipelas in small pig houses may be associated with the use of straw as bedding, unpaved paddocks, and insufficient removal of manure, which may be sources of *E. rhusiopathiae* [3]. Erysipelas is less common in large piggeries with a closed production cycle, equipped with structures for automatic removal of animal excrement. However, it sometimes occurs even in single vaccinated pigs bred on farms with proper biosecurity rules. The onset of the disease in such cases may be the result of human error during vaccination (inappropriate vaccine handling), or individual characteristics associated with the failure to develop an immune response following immunization [31,32]. It should also be considered that commercial vaccines may not be fully effective against field strains representing a different serotype and variant Spa antigen than the vaccine strains. In addition, field strains may differ from vaccine strains in terms of other surface antigens. The cases of erysipelas in vaccinated pigs reported in this study were caused by strains of serotypes 2 (5S, 9S), 6 (2S) and 8 (8S). A case of infection in a vaccinated pig caused by a SpaA-positive strain of serotype 6 was also reported by Shimoji et al. [33]. The results presented in this paper may be important in epidemiological analyses and in the selection of epidemiologically relevant candidates for vaccines.

### 4.2. Serotypes of E. rhusiopathiae Strains

The high prevalence of *E. rhusiopathiae* serotype 1b strains (42.8%) and lower prevalence of other serotypes, i.e., 2, 5, 6, 8 and N (7.1–21.4%), in this study is partly consistent with literature reports [34,35]. In other countries, *E. rhusiopathiae* serotypes 1 and 2 are frequently isolated from clinically affected pigs and have the highest prevalence and economic importance [36]. McNeil et al. [34] demonstrated serotype 2 dominance (58.6%) in *E. rhusiopathiae* isolates from pigs in the UK (n = 128, strains collected between 1987 and 2015); less frequently, serotypes 1a (16.4%) and 1b (13.3%) were identified, and very rarely other serotypes, including 5 (1.6%) and N (3.1%) [34]. In Japan, among *E. rhusiopathiae* strains (n = 800) isolated from swine erysipelas (between 1992 and 2002), serotypes 1a (47.6%), 2b (31.7%) and 1b (18.25%) were most common [35]. Results significantly different from ours were also obtained in Germany, where serotype 1a (62.5%) dominated among strains (n = 32) from birds and mammals, while strains representing serotype 2 (28.1%) and serotype N (9.4%) were found less frequently [16].

Serotyping may be helpful in epidemiological studies to assess the spread of strains in the environment, especially locally between farms. Several studies have shown that immunization of pigs with inactivated (bacterin) or attenuated serotype 2 strains confers immunity against serotype 2 and 1 strains and, to varying degrees, against strains of other serotypes, including 5, 6, 8, and N, detected in this study [37,38,39]. Significant variation in cross-protection was noted in an experiment using mice. Animals vaccinated with attenuated strain Koganei 65–0.15 serotype 2 survived when challenged with serotypes 1b, 2, 8, and type N, but mortality occurred in mice exposed to serotypes 1a, 11, 12, 15, 16, or 21 (20–30% mortality), 4, 5, 6, 7, or 8 (40–50% mortality), and 9, 10, 18, or 19 (60–80% mortality) [38]. However, Forde et al. [36] suggest a cautious approach to the results of these provocation studies since they included only one representative strain of serotype 2 and that the results may have been influenced by immunogenic characteristics other than the serotype. It seems, therefore, that the question of whether the serotype is an important feature determining cross-protection requires more thorough research.

### 4.3. Antibiotic Susceptibility and Genotypic Resistance Profiles

The widespread susceptibility of *E. rhusiopathiae* strains to ampicillin and ceftiofur demonstrated in this study is fully consistent with several previous reports [1,11,28,40,41]. It is surprising that, in the era of rapidly increasing drug resistance among bacteria and the high use of beta-lactam antibiotics in animal husbandry [42], strains of *E. rhusiopathiae* isolated in various geographic regions of the world are still susceptible to this group of antimicrobial substances. This is good news for veterinarians and farmers, as penicillin is currently the drug of choice for swine erysipelas [2]. In pig medicine in Poland, amoxicillin and amoxicillin with clavulanic acid can also be used, as well as two other antimicrobial substances to which all *E. rhusiopathiae* strains were sensitive (tylosin and florfenicol). The reported susceptibility to florfenicol is consistent with the previous studies by Fidalgo et al. [43], who showed the MIC of chloramphenicol in *E. rhusiopathiae* strains (n = 60) ranged from 8 to 16 µg/mL. The widespread susceptibility of *E. rhusiopathiae* isolates to macrolides noted in this study is in contrast to the results of Wu et al. [28], who showed that as many as 53.3% of strains from pigs in China (collected between 2012 and 2018) were resistant to erythromycin. Interestingly, Ding et al. [1], examining isolates also collected in China (between 2012 and 2013), did not record erythromycin MIC values exceeding 1 µg/mL in any strain (all tested isolates were sensitive). 

According to the adopted criteria, only 1 of 14 (7.1%) of the tested isolates was resistant to lincomycin, clindamycin, and tiamulin. Previously, widespread susceptibility to tiamulin (≤6.25 µg/mL) was recorded in *E. rhusiopathiae* strains isolated from pigs in Japan [41] and in Brazil [40]. The *lsaE* resistance gene detected in a tiamulin-resistant strain has previously been identified as part of plasmid-borne or chromosomal multi-drug resistance gene clusters in other gram-positive bacteria [44,45], and has recently been found in *E. rhusiopathiae* strains from pigs [28]. The results obtained in this work are consistent with previous reports from Japan showing that the vast majority of *E. rhusiopathiae* strains (obtained between 1988 and 1998) were susceptible to lincomycin and clindamycin [41]. Completely opposite results were recorded in China, where the percentage of strains resistant to lincosamides ranged from 64% to 72% [1,28]. The presence of the *lincosamide* nucleotidyltransferase *lnuB* gene in a phenotypically-resistant strain has previously been reported in various gram-positive bacteria from pigs, including *E. rhusiopathiae* [46,47,48]. 

The prevalence of tetracycline-resistant strains (71.4%) reported in this study is higher than that reported in China and Japan, where 50.8–60.4% of *E. rhusiopathiae* strains were tetracycline-resistant and 38% were doxycycline-resistant [1,28,41]. The reason for such a high prevalence of tetracycline-resistant strains may be the frequent use of tetracyclines in pig farming in Poland [49]. The correlation observed between tetracycline resistance and the presence of the *tetM* gene is consistent with the findings of Wu et al. [28], who showed the presence of *tetM* in 57.4% of tetracycline-resistant strains. The coexistence of the *tetM* gene and the transposon integrase Tn916 indicates the involvement of this mobile genomic element in the spread of the *tetM* gene in *E. rhusiopathiae* strains.

The percentage of strains resistant to enrofloxacin (57.1%) reported in this study is similar to the result obtained by Ding et al. [1] in China (~70% of *E. rhusiopathiae* strains showed resistance to norfloxacin and levofloxacin), but much lower than the percentage of fluoroquinolone-resistant *E. rhusiopathiae* strains recorded in other studies [28,40]. The reported mechanism of resistance associated with the mutation at position 86 (Thr86→Ile) of the GyrA subunit of DNA gyrase is consistent with the previous findings of Wu et al. [28]. However, it should be noted that these authors also reported mutations at position 90 of GyrA and position 82 of the ParC subunit of DNA topoisomerase IV in *E. rhusiopathiae* strains resistant to ciprofloxacin [28]. 

The high MICs of gentamicin, kanamycin, neomycin, trimethoprim, trimethoprim/sulfadiazine, and rifampicin reported in this study, together with the unimodal MIC distribution and the absence of resistance genes, indicate that *E. rhusiopathiae* strains are inherently resistant to these antimicrobials. Wild unimodal MIC distributions were noted for streptomycin and spectinomycin, but their MIC values were lower than those of the other aminoglycosides. Interestingly, the *aadK* gene, that encodes aminoglycoside 6-adenylyltransferase, the streptomycin-modifying enzyme, was detected in all strains tested. It should be noted, however, that the sequence of the *aadK* gene of the Fujisawa strain (GenBank Acc. No. AP012027.1, locus_tag=“ERH_1545) is not homologous with the sequence of the *aadK* gene found in other bacteria, i.e., in the strain *Bacillus subtilis* 168 (Acc. No. NG_047379.1) [50] and in strain 4300STDY6542365 *Klebsiella pneumoniae* (Acc. No. UFEG01000012.1) (data not shown, obtained from BLAST analysis). The widespread occurrence of high MIC values of kanamycin (MIC >100 µg/mL) in *E. rhusiopathiae* strains has also been reported by other authors [1,11,41]. However, the gentamicin susceptibility results obtained in this study differ slightly from those of Ding et al. [1], who demonstrated a wide range of gentamicin MICs (2–>128 µg/mL) among *E. rhusiopathiae* strains from China. The resistance of *E. rhusiopathiae* strains to folic acid inhibitors (trimethoprim, trimethoprim/sulfadiazine) is consistent with the results of previous studies [1,11,40]. The high rifampicin MIC values (MIC >128 µg/mL) indicative of intrinsic resistance have not been previously reported in *E. rhusiopathiae*. Other gram-positive bacteria are generally susceptible (MIC ≤ 2 µg/mL) to this RNA polymerase inhibitor [51,52].

### 4.4. Virulence Genes

The common occurrence of the *spaA* gene in *E. rhusiopathiae* isolates recorded in this study is consistent with the results of other studies on both porcine and poultry strains [16,34]. The exclusive discovery of *spaA* and the lack of *spaB* and *spaC* in the collected *E. rhusiopathiae* isolates may be because the strains belong to serotypes for which the presence of *spaA* is characteristic (1b, 2, 5, and 8) [17]. The presence of the *spaA* gene was recorded even in the 2S strain of serotype 6, although earlier reports showed that strains of this serotype are usually *spaB*-positive [17]. The prevalence of other selected potential virulence genes in *E. rhusiopathiae* strains noted in this work is also consistent with several other reports [1,16,53]. Janßen et al. [16] demonstrated the presence of the genes *nanH.1*, ERH_1356, *intl-like*, *rspA*, *rspB*, *algI*, *sub*, *hlyA*, *fbpA*, and *hlyIII* in all tested *E. rhusiopathiae* strains (n = 165). Only the *intl* gene encoding internalin-like protein was absent in 15% of the analysed isolates, primarily from poultry and sheep. Zhu et al. [15] showed that the product of the *intI* gene (ERH_1472) acts as an adhesin enabling specific adherence of *E. rhusiopathiae* to the surface of pig iliac arterial endothelial cells. In *Listeria monocytogenes*, internalin contributes to the invasion of the bacteria into epithelial cells [54].

A study based on analysis of whole genome sequences of eight virulent strains of *E. rhusiopathiae* (Fujisawa, NCTC8163/ACTC 19414, WH13013, ZJ, ML101, GXBY-1, SY1027, and KC-Sb-R1) showed that genes encoding enzymes involved in synthesis of the bacterial capsule (*cpsA*, *cpsB*, and *cpsC*), neuraminidase (*nanH*), hyaluronidase (*hylA*, *hylB*, and *hylC*), and surface proteins (*spaA*, *rspA*, and *rspB*) are core genes [53]. The results of our study differ somewhat from those of Ding et al. [1], who failed to detect the *hylA* (ERH-0150), *nanH.1*, and ERH-1356 gene in 8.3%, 6.25%, and 22.9% of *E. rhusiopathiae* strains from pigs, respectively. However, the negative result was probably due to the fact that the primers designed by these authors were complementary to the sequences outside these genes (this conclusion is based on the BLAST analysis of primer annealing sites to the sequence of the Fujisawa strain, Acc. No. AP012027.1). The reported discrepancies may also be due to the small number of strains tested in these studies.

It should be emphasized that the role of only some of the putative virulence genes detected in this work has been confirmed in the pathogenesis of erysipelas. Zhu et al. [22], in a study with recombinant *E. rhusiopathiae* SpaA (rSpaA), demonstrated that this protein adheres to porcine endothelial cells, and its plasminogen-binding activity is highly likely to play a role in this adhesion. Li et al. [29], based on comparative analyses of gene expression in the highly virulent HX130709 and its isogenic avirulent derivative HX130709a, showed that SpaA and neuraminidase are key virulence factors of *E. rhusiopathiae*. Earlier research by Shimoji et al. [55] demonstrated that the virulence of *E. rhusiopathiae* is largely dependent on the presence of the capsular antigen, but not on hyaluronidase. Literature reports on the role of RspA and RspB proteins in the pathogenesis of *E. rhusiopathiae* infections are ambiguous. Initially, they indicated that these proteins are exposed on the cell surface of *E. rhusiopathiae* and participate in biofilm formation. Moreover, recombinant RspA, but not RspB, elicitated protection in mice against experimental challenge [56]. However, the results of a recent study by Li et al. [29] indicate no relationship between RspA and RspB and the virulence of *E. rhusiopathiae*. 

### 4.5. SpaA Variants

Several authors have studied *spaA* gene sequences in *E. rhusiopathiae* strains, analysing nonsynonymous mutations in the region corresponding to the N-terminal immunoprotective domain (corresponding to 30–413 aa) and the number of tandem repeats in the C-terminal segment of the gene (corresponding to 448–626 aa in SpaA proteins containing 9 tandem repetitions) [16,17,19]. The 100% sequence homology between isolates in the signal region of SpaA (1–29 aa) reported in this work is consistent with previous findings [16,17].

Our results of analyses of the C-terminal region of SpaA, that is responsible for binding the protein to the bacterial cell surface, are consistent with the research of Janßen et al. [16], who showed that SpaA of *E. rhusiopathiae* strains from poultry and pigs in Germany most often contains 9 tandem repeats (89.7% strains), but their number varies from 7 to 13. In this work, the relationship between the number of tandem repeats and the serotype was demonstrated for the first time (8 tandems were found only in strains of serotype 1b, and 7 tandems were specific for serotype 8). A recent study by Wu et al. [57] showed that the number of tandem repeats in the SpaA chain affects the adhesive properties of *E. rhusiopathiae*. The *ΔspaA* mutant strain (mutated SE38 strain virulent in pigs) producing the SpaA protein truncated by 2 tandem repeats (120 nt deletion) displayed attenuated virulence in mice and decreased adhesion to porcine endothelial cells [57]. Thus, the strains tested in this work that have 7 or 8 tandems in the C-terminal region of SpaA can be expected to be less virulent than strains producing SpaA with 9 repeats.

Based on polymorphisms in the N-terminal 447-aa region of SpaA, including a 384-aa hypervariable region (30–413 aa) and a 34-aa proline-rich region (414–447 aa), the 14 isolates were classified into 7 groups. The aa substitutions recorded at positions 55, 70, 101, 178, 195, 257, and 303 of the SpaA protein chain were previously observed in *E. rhusiopathiae* strains [16,19], while the polymorphisms at positions 38, 54, 109, 139, and 232 (hypervariable domain) and 423, 426, and 435 (proline-rich domain) have not previously been described. The His109 mutation was unique to the R32E11 vaccine strain. Janßen et al. [16], based on the aa substitutions in the N-terminal protective region of SpaA, divided the strains tested (n = 165) into five groups (I–V). However, in contrast to our results, these authors did not observe a relationship between the serotype and the SpaA variant [16]. Most of our strains (9/14, 64.2%) (serotype 1b, 2 and 5) correspond to their group II SpaA (Ser101 and Ile257). However, unlike the prevalence of this SpaA variant in our strains, Janßen et al. [16] included only one of 36 isolates (2.8%) from pigs and 21.1% of isolates from other hosts, mainly chickens and turkeys, in group II. The SpaA sequence of the 14S strain (Ile55, Asn70, Asp178, Asn195, Ile257 and Glu303) is homologous with the sequence of the reference strain ATCC 19414 and with SpaA variant I, to which Janßen et al. [16] assigned the majority (52.7%) of tested strains, including 18 (50%) of 36 from pigs. It should be noted that Janßen et al. [16] assigned an incorrect aa to the GAG codon, which resulted in the Glu303 polymorphism being designated Gln303. The SpaA sequence of our strain 2S serotype 6 is homologous to SpaA variant III (Ile257) distinguished by Janßen et al. [16]; this SpaA variant, unique to our isolates, was found in 22.4% of *E. rhusiopathiae* strains tested in Germany, including 13 of 36 porcine strains (36.1%). The strains 1S, 8S, and 12S represent new, as yet undescribed, variants of SpaA; however, the high homology of the 1S strain with group I distinguished by Janßen et al. [16] should be noted (the difference concerns only Gln38). Among isolates (n = 34) from pigs in Japan, three SpaA variants were distinguished, based on substitutions at aa positions 195, 203, and 257 [19].

Sequence analyses of the SpaA of the R32E11 vaccine strain against the sequence of the wild-type *E. rhusiopathiae* strains have thus far not been performed. Of the 14 pig isolates, none showed 100% homology to the sequence of the immunoprotective domain of strain R32E11 (differences ranged from 1 to 8 aa). It is also worth mentioning that the number of tandem repeats in the C-terminal section of SpaA in this strain (13) is much higher than in field isolates (7–9). It is not known whether the structural variations in the SpaA immunogen of the R32E11 strain translate into a protective effect of the vaccine against infections with field *E. rhusiopathiae* strains. Only studies in animal models could clarify this issue.

### 4.6. Limitations of the Study

The main limitation of the current study was the small sample size. The analyses carried out on 14 strains, do not give a full view of the antibiotic susceptibility, serotypes or SpaA variants of E. rhusiopathiae strains causing swine erysipelas in Poland despite the fact that they came from three regions of the country. There is a need for further research in this area on a larger scale. We also regret that, due to the lack of public access to the strains based on which vaccines against erysipelas are prepared, we could not include them in the comparative analyses. It should be noted that the DNA of the R32E11 strain was not isolated directly from the vaccine (previous attempts to isolate DNA from the ERYSENG^®^, Hipra, vaccine failed [36]), but from the E. rhusiopathiae R32E11 strain deposited in the collection of the National Institute of Animal Health in Japan.

## 5. Conclusions

The research results presented in this paper are the first reports on the occurrence of porcine erysipelas in Poland and the characteristics of *E. rhusiopathiae* strains. Despite the use of a small number of samples, we have shown that *E. rhusiopathiae* strains causing swine erysipelas in Poland are diverse in terms of antibiotic susceptibility, serotypes, and variants of the SpaA immunogen. Analyses involving the R32E11 vaccine strain provided valuable information on its relationship with circulating field strains and created space for possible research on the development of a new swine erysipelas vaccine. Due to the lack of diversity of *E. rhusiopathiae* strains in terms of the occurrence of potential virulence genes, their detection does not provide significant information on the virulence of these bacteria. Assessment of the participation of individual genes in the pathogenesis of erysipelas requires more in-depth research based on gene expression analysis or the use of knockout strains.

Our research shows that in the treatment of swine erysipelas in Poland, apart from the commonly used beta-lactam antibiotics, macrolides, and phenicols, can also be effective. Aminoglycoside antibiotics, folic acid inhibitors, tetracyclines, and fluoroquinolones should not be considered. The high prevalence of resistance to enrofloxacin and tetracycline indicates the need to limit the use of these antibiotics in pig farming in Poland. 

## Figures and Tables

**Table 1:** MIC (µg/mL) breakpoints used to categorize *E. rhusiopathiae* strains as susceptible (S), intermediate (I), and resistant (R) in the broth microdilution method.

Antimicrobial Agent	Breakpoints			Reference
	S	I	R	
Ampicillin	≤0.25	–	–	CLSI VET06 [26]
Ceftiofur	≤2	4	≥8	CLSI VET06 [26]
Erythromycin	≤0.25	0.5	≥1	CLSI VET06 [26]
Tylosin	≤0.25	0.5	≥1	CLSI VET06 breakpoints for erythromycin were adopted [26].
Clindamycin	≤0.25	0.5	≥1	CLSI VET06 [26]
Lincomycin	≤2	4–8	≥16	CA-SFM/EUCAST breakpoints for *Staphylococcus* spp. were adopted [27]
Tiamulin	≤16	–	≥32	Breakpoints were proposed based on MIC distribution and the presence of the *lsaE* gene
Enrofloxacin	≤0.5	1	≥2	CLSI VET06 [26]
Tetracycline	≤4	8	≥16	CLSI breakpoints for *Staphylococcus* spp. were adopted [25]; a breakpoint of ≥16 µg/mL has also been previously proposed for *E. rhusiopathiae* by other authors [1,28]
Florfenicol	≤8	16	≥32	Chloramphenicol cut-offs recommended by CLSI [25] for *Staphylococcus* spp. and *Enterobacteriaceae* have been adopted

**Table 2:** MIC values of 17 antimicrobial substances obtained in the *E. rhusiopathiae* susceptibility test and detected resistance genes. MIC values highlighted in grey indicate resistance.

Strain ID	Ser.	AMP	CEF	TET	ERY	TYL	LIN	CLI	TIA	ENR	FLO	STR	SPE	GEN	KAN	NEO	TR	TR/S	RIF	Resistance Genes
ATCC 19414	2	≤0.06	≤0.06	0.5	0.25	0.125	0.5	≤0.06	4	≤0.25	2	128	64	>512	>512	>512	>512	>512	>128	*aadK*
1S	N	≤0.06	≤0.06	1	0.125	0.125	1	≤0.06	2	≤0.25	4	32	32	>512	>512	>512	>512	>512	>128	*aadK*
2S	6	≤0.06	≤0.06	0.5	0.125	≤0.06	0.5	≤0.06	2	≤0.25	2	64	16	>512	>512	>512	>512	>512	>128	*aadK*
3S	1b	0.125	≤0.06	32	0.25	0.125	0.5	≤0.06	4	16	4	128	32	>512	>512	>512	>512	>512	>128	*aadK*, *tetM*, *int-Tn*
4S	1b	0.125	≤0.06	64	0.5	0.125	0.5	≤0.06	4	16	4	256	128	>512	>512	>512	>512	>512	>128	*aadK*, *tetM*, *int-Tn*
5S	2	0.125	≤0.06	32	0.25	0.125	1	≤0.06	4	≤0.25	4	256	64	>512	>512	>512	>512	>512	>128	*aadK*, *tetM*, *int-Tn*
6S	5	0.125	≤0.06	32	0.25	0.125	0.5	0.25	2	8	4	32	64	>512	>512	>512	>512	>512	>128	*aadK*, *tetM*, *int-Tn*
7S	1b	0.125	≤0.06	32	0.25	0.125	0.5	≤0.06	2	16	2	256	128	>512	>512	>512	>512	>512	>128	*aadK*, *tetM*, *int-Tn*
8S	8	0.125	≤0.06	32	0.25	0.25	>64	2	>128	16	4	128	64	>512	>512	>512	>512	>512	>128	*aadK*, *tetM*, *int-Tn*, *lnuB*, *lsaE*
9S	2	≤0.06	≤0.06	32	≤0.06	0.25	0.5	≤0.06	4	≤0.25	2	256	32	>512	>512	>512	>512	>512	>128	*aadK*, *tetM*, *int-Tn*
10S	5	≤0.06	≤0.06	64	0.25	0.125	0.5	≤0.06	4	8	2	256	32	>512	>512	>512	>512	>512	>128	*aadK*, *tetM*, *int-Tn*
11S	1b	0.25	≤0.06	64	0.25	0.125	0.5	≤0.06	4	16	2	128	64	>512	>512	>512	>512	>512	>128	*aadK*, *tetM*, *int-Tn*
12S	1b	≤0.06	≤0.06	0.125	0.25	0.125	0.5	≤0.06	4	≤0.25	4	32	64	>512	>512	>512	>512	>512	>128	*aadK*
13S	1b	0.125	≤0.06	64	0.125	0.125	0.5	≤0.06	4	16	2	128	32	>512	>512	>512	>512	>512	>128	*aadK*, *tetM*, *int-Tn*
14S	2	≤0.06	≤0.06	0.5	≤0.06	≤0.06	0.125	≤0.06	1	≤0.25	4	32	32	>512	>512	>512	>512	>512	>128	*aadK*
Number and % of resistant strains		0	0	10 71.4%	0	0	1 7.1%	1 7.1%	1 7.1%	8 57.1%	0	NA	NA	NA	NA	NA	NA	NA	NA	

Legend: Ser.—serotype; AMP—ampicillin; CEF—ceftiofur; TET—tetracycline; ERY—erythromycin; TYL—tylosin; CLI—clindamycin; LIN—lincomycin; TIA—tiamulin; ENR—enrofloxacin; FLO—florfenicol; STR—streptomycin; SPE—spectinomycin; GEN—gentamicin; KAN—kanamycin; NEO—neomycin; TR—trimethoprim; TR/S—trimethoprim/sulfadiazine; RIF—rifampicin; NA–not applicable.

**Table 3:** Sequence analysis of the quinolone resistance determining region (QRDR) in the *gyrA* gene in enrofloxacin susceptible and resistant *E. rhusiopathiae* strains.

Isolate	Serotype	Enrofloxacin MIC [µg/mL]	Antibiotic Susceptibility	Mutation at Position 257 of the *gyrA* Gene and Corresponding Change in aa Sequence	GenBank Accesion NUMER
ATCC 19414	2	≤0.25	S	Thr_86_ (ACA)	LR134439.1
1S	N	≤0.25	S	Thr_86_ (ACA)	OP921301
2S	6	≤0.25	S	Thr_86_ (ACA)	OP921302
3S	1b	16	R	Thr_86_→Ile (ACA→ATA)	OP921303
8S	8	16	R	Thr_86_→Ile (ACA→ATA)	OP921304
10S	5	8	R	Thr_86_→Lys (ACA→AAA)	OP921305

Legend: S—susceptible, R—resistant.

**Table 4:** Prevalence of potential genes associated with virulence in the tested strains of *E. rhusiopathiae*.

Gene/Locus Tag Acc. No. AP012027.1	Gene Product/Predicted Function	Gene Prevalence
*spa;* ERH_0094	Surface protection antigen A/adhesion to host cells	100%
*spaB*	Surface protection antigen B	0%
*spaC*	Surface protection antigen C	0%
*nanH.1*; ERH_0299	Neuraminidase/spreading factor	100%
*nanH.2*; ERH-0761	Neuraminidase/spreading factor	100%
*cpsA*; ERH_0157	Capsule polysaccharide synthesis gene (glycosyl transferase)/resistance to complement	100%
ERH_1356	ABC transporter metal-binding lipoprotein/adhesion of host cells	100%
*intl*; ERH_1472	Internalin/invasion of epithelial cells	100%
*rspA*; ERH_0668	Rhusiopathiae surface protein/biofilm formation	100%
*rspB*; ERH_0669	Rhusiopathiae surface protein/biofilm formation	100%
*algI*; ERH_0402	Alginate-O-acetyltransferase/resistance to phagocytosis	100%
*sub*; ERH_0260	Cell-envelope associated proteinase, subtilase family	100%
*hlyA*; ERH_0150	Hyaluronidase/adhesion-promoting factor	100%
*fbpA*; ERH_1034	Fibronectin-binding protein/adhesion	100%
*hlyIII*; ERH_0649	Haemolysin/lytic activity on red blood cells	100%

**Table 5:** Nonsynonymous mutations in the N-terminal hypervariable and proline-rich region of the *spaA* gene and number of C-terminal tandem repeats in the wild-type *E. rhusiopathiae* strains compared with the corresponding sequence of the *E. rhusiopathiae* R32E11 vaccine strain as well as the Fujisawa and ATCC 19414 reference strains.

SpaA Variant		Serotype	Amino Acid (aa) Position															Number of C-Terminal Tandem Repeats
			38	54	55	70	101	109	139	178	195	232	257	303	423	426	435	
	R32E11 vaccine strain	2	CCA (Pro)	GGG (Gly)	ATA (Ile)	AAT (Asn)	AAC (Asn)	CAT (His)	CAG (Gln)	GAT (Asp)	AAT (Asn)	ATC (Ile)	ATT (Ile)	GAG (Glu)	CCA (Pro)	AAA (Lys)	CCA (Pro)	13
	Fujisawa type strain	1a			GTA (Val)	AAA (Lys)		AAT (Asn)		GGT (Gly)	GAT (Asp)		CTT (Leu)	GGG (Gly)		GAA (Glu)	CTA (Leu)	9
	ATCC 19414 type strain	2						AAT (Asn)										9
1	14S	2						AAT (Asn)										9
2	1S	N	CAA (Gln)					AAT (Asn)										9
3	2S	6			GTA (Val)	AAA (Lys)		AAT (Asn)		GGT (Gly)	GAT (Asp)			GGG (Gly)				9
4	8S	8		GCG (Ala)	GTA (Val)	AAA (Lys)	AGC (Ser)	AAT (Asn)		GGT (Gly)	GAT (Asp)			GGG (Gly)				7
5	12S	1b			GTA (Val)	AAA (Lys)	AGC (Ser)	AAT (Asn)	AAG (Lys)	GGT (Gly)	GAT (Asp)	ACC (Thr)		GGG (Gly)				8
6	3S, 4S, 6S, 7S, 10S, 11S, 13S	1b, 5			GTA (Val)	AAA (Lys)	AGC (Ser)	AAT (Asn)		GGT (Gly)	GAT (Asp)			GGG (Gly)				8 or 9
7	5S, 9S	2			GTA (Val)	AAA (Lys)	AGC (Ser)	AAT (Asn)		GGT (Gly)	GAT (Asp)			GGG (Gly)	CAA (Gln)			9

## Data Availability

Nucleotide sequences reported in this paper have been deposited in the NCBI GenBank database under the following accession numbers: OP921301-OP921305 (*gyrA*), OP822679-OP822691, OQ054982 and MZ448116 (*spaA*).

## Supplementary Materials

The following supporting information [51,58,59,60,61,62,63] can be downloaded at: https://www.mdpi.com/article/10.3390/pathogens12030409/s1, Table S1. Vaccines against swine erysipelas available in Poland; Table S2. Information on pigs from which *E. rhusiopathiae* strains were isolated; Table S3. Primers used for serotyping of *E. rhusiopathiae* strains; Table S4. Primers used for detection of antimicrobial resistance genes in *E. rhusiopathiae*; Table S5. Bacterial strains used as positive controls for the detection of resistance genes; Table S6. Primers used for detection of virulence-associated genes in *E. rhusiopathiae* strains; Table S7. List of *E. rhusiopathiae* strains whose *spaA* gene sequences were used for comparative analysis and clustering; Figure S1. (a,b) Skin lesions seen in some pigs from which *E. rhusiopathiae* strains were isolated; (c) May Grunwald Giemsa-stained blood smear of pig No. 14—in the centre there are visible slender rods resembling *E. rhusiopathiae* (magnification 1000×); (d) Culture of *E. rhusiopathiae* on blood agar; (e) alpha-haemolytic activity of representative *E. rhusiopathiae* strain (10× magnification); (f) *E. rhusiopathiae* morphology visualised using Gram staining (magnification 1000×, Olympus DP72 microscope).
