# Supplementary material for: Serotypes, Antibiotic Susceptibility, Genotypic Virulence Profiles and SpaA Variants of Erysipelothrix rhusiopathiae Strains Isolated from Pigs in Poland
Source: Pathogens. 2023 Mar 3;12(3):409. doi: 10.3390/pathogens12030409 (PMC10058941; doi:10.3390/pathogens12030409)
Supplement: Supplementary file 1 [file pathogens-12-00409-s001.zip › pathogens-2164744-supplementary.pdf]

Supplement

Table S1. Vaccines against swine erysipelas available in Poland.

Name	Vaccine type	*E. rhusiopathiae* strains and their serotypes [according to the manufacturer's leaflet]	Producer
Parvoerysin	inactivated	2-64 serotype 2a;<br>2-5 serotype 2a;<br>2-II serotype 2a;<br>1-203 serotype 1a	Bioveta, Czech Republic
Erysin Single Shot	inactivated	2-64 serotype 2a;<br>2-5 serotype 2a;<br>2-II serotype 2a;<br>1-203 serotype 1a	Bioveta, Czech Republic
Ruvax	inactivated	*E. rhusiopathiae* serotype 2	Merial S.A.S., Boehringer-Ingelheim Vetmedica, France
Parvoruvax	inactivated	*E. rhusiopathiae* serotype 2	CEVA-Phylaxia Veterinary Biologicals Co. Ltd., Hungary
ERYSENG®	inactivated	R32E11	Laboratorios Hipra S.A., Spain
ERYSENG® PARVO	inactivated	R32E11	Laboratorios Hipra S.A., Spain
Porcilis Ery + Parvo+ Lepto	inactivated	M2, serotype 2	Intervet International B.V., Netherlands
Porcilis Ery	inactivated	M2, serotype 2	Intervet International B.V., Netherlands
Suibiovac Ery	inactivated	OV1, serotype 1<br>OV2, serotype 2	Drwalewskie Zakłady Przemysłu Bioweterynaryjnego S.A., Poland

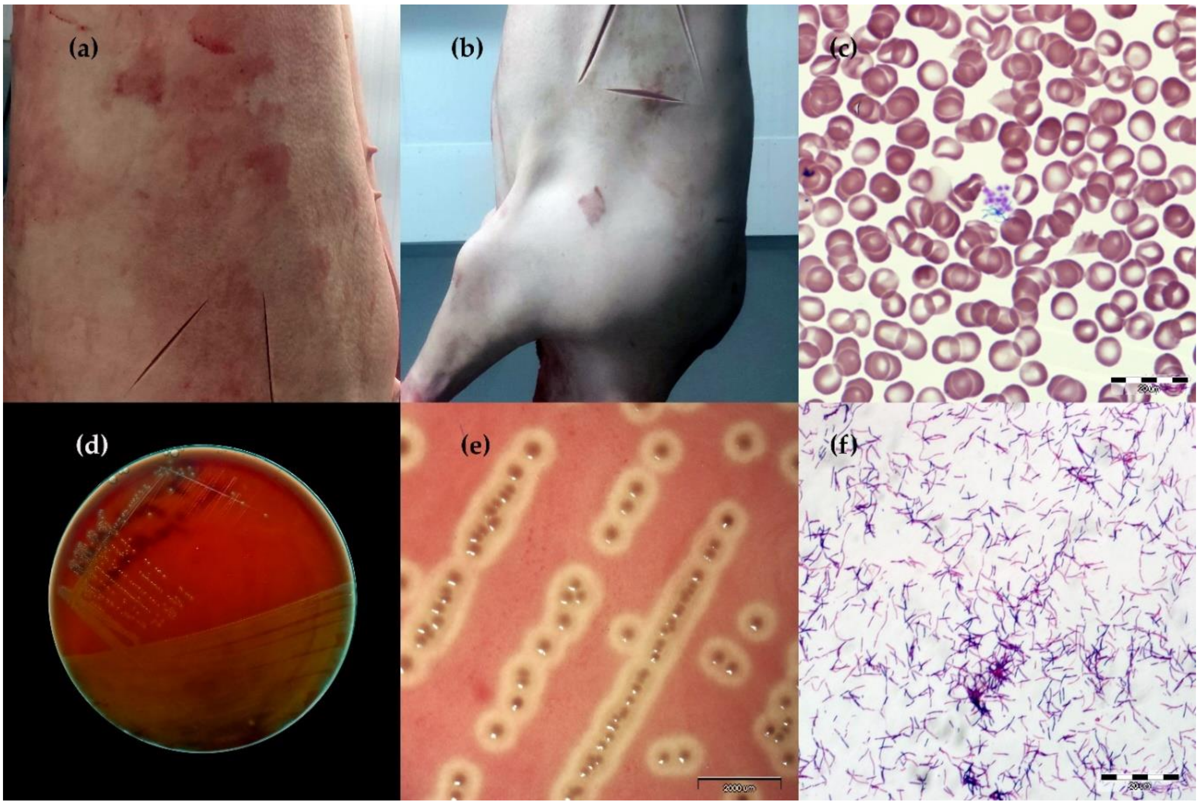

Figure S1. a) and b) Skin lesions seen in some pigs from which *E. rhusiopathiae* strains were isolated; c) May Grunwald Giemsa-stained blood smear of pig No. 14 – in the centre there are visible slender rods resembling *E. rhusiopathiae* (magnification 1000x); d) Culture of *E. rhusiopathiae* on blood agar; e) alpha-haemolytic activity of representative *E. rhusiopathiae* strain (10x magnification); f) *E. rhusiopathiae* morphology visualised using Gram staining (magnification 1000x, Olympus DP72 microscope).

Table S2. Information on pigs from which *E. rhusiopathie* strains have been isolated.

Pig ID	Strain ID	Sero-type	Year of isolation	Clinical signs/history of disease	Tissues from which *E. rhusiopathiae* was isolated	Vaccination status	Age of pig [months]	Town/ province
1	1S	N	2017	the animal showed no clinical signs of disease; in the post-mortem inspection a thickening in the subcutaneous tissue was found; the pig had previously been treated with antibiotics for fever and skin lesions	sections of altered subcutaneous tissue	unvaccinated	7	Frankamionka/ Lubelskie
2	2S	6	2018	skin lesions and thickening in the subcutaneous tissue was observed	sections of altered subcutaneous tissue	vaccinated	4	Chojnice/ Pomorskie
3	3S	1b	2018	the animal got up less often than other animals; according to the owner, there have been no confirmed cases of erysipelas on the farm before	sections of altered subcutaneous tissue, tonsils	unvaccinated	7	Gdeszyn/ Lubelskie
4	4S	1b	2019	the animal showed no clinical signs of disease; in the post-mortem inspection a thickening in the subcutaneous tissue was found; there were previous cases of erysipelas in other animals on the farm, emergency treatment was used	sections of altered subcutaneous tissue, altered heart valves	unvaccinated	9	Gdeszyn / Lubelskie
5	5S	2	2019	the animal showed no clinical signs of disease; in the post-mortem inspection a thickening in the subcutaneous tissue was found;	sections of altered subcutaneous tissue	vaccinated	5	Chojnice/ Pomorskie
6	6S	5	2019	the animal showed no clinical signs of disease; in the post-mortem inspection a thickening in the subcutaneous tissue was found; the pigs had previously been treated with antibiotics for fever and reluctance to move and eat	sections of altered subcutaneous tissue	unvaccinated	9	Dobromierzyce/ Lubelskie
7	7S	1b	2019	the animal showed no clinical signs of disease; in the post-mortem inspection a thickening in the subcutaneous tissue was found; previous antibiotic treatment due to problems with the hind limbs; there were previous cases of erysipelas on the farm	fragment of skin with subcutaneous tissue and muscle fragment	unvaccinated	8	Chojnice/ Pomorskie

8	8S	8	2019	in the post-mortem inspection a thickening in the subcutaneous tissue was found	fragment of the altered muscular fascia	vaccinated	5	Chojnice/ Pomorskie
9	9S	2	2019	the animal showed no clinical signs of disease; in the post-mortem inspection a thickening in the subcutaneous tissue was found;	sections of altered subcutaneous tissue and skin	vaccinated	5	Chojnice/ Pomorskie
10	10S	5	2018	the pig had previously been treated with antibiotics due to problems with the hind limbs; there were previous cases of erysipelas on the farm	contents and fragments of the cartilages of the hock joints	unvaccinated	7	Horyszów/ Lubelskie
11	11S	1b	2017	no information available	synovial fluid	unvaccinated	7	Grabowiec/ Lubelskie
12	12S	1b	2018	the animals showed no clinical signs of disease; in the post-mortem inspection a thickening in the subcutaneous tissue was found	a single red skin lesion in the chest area along with subcutaneous tissue	unvaccinated	10	Chojnice/ Pomorskie
13	13S	1b	2018	the animals showed no clinical signs of disease; in the post-mortem inspection a thickening in the subcutaneous tissue was found; the animals were previously treated with penicillin for fever and red skin lesions; there were previous cases of erysipelas on this farm	tonsils and a fragment of the heart with valves	unvaccinated	7	Gdeszyn/ Lubelskie
14	14S	2	2022	skin lesions have been observed	fragment of a skin lesion, blood sample	unvaccinated	6	Dortka/ Świętokrzyskie

Table S3. Primers used for serotyping of *E. rhusiopathiae* strains.

	Serotype	Sequence of primers (5' to 3')	Annealing temp. [°C]	Size of PCR product [bp]	Reference
Multiplex I	1a	1a-F: CTCCTAACGCTTTAGCACGC<br>1a-R: TGA TCC TTT GCC ACT AAT GC	60	356	[24]
	1b	1b-F: CGAAAGCATCCCTGTAATCAGTTGC<br>1b-R: TGCCTGTAAAACCTGATCGTCTAAATC		1357	
	2	2F: CCACGTCTTCCCACACTACAAAAAAGTAAATTC<br>2R: TCATCCTAATGCATATCATTATGTGCATATGAA		541	
	5	5F: GCACGTTTCCAAATATTGTATCGAGTCT<br>5R: GAAATAATGCCGATAGATGGAGCACC		194	
Multiplex II	6	6F: CAAGGCTTGCGCGTTTGGAC<br>6R: TTCATGGCATGGTGGTGGCG	60	573	[7]
	8	8F: AGCAATAAGATTGTAGATTAGCCAA<br>8R: TACCTTCCATCTAGGATAATGAAGG		474	
	9	9F: AGTTCCTGCAGAAACGCCTT<br>9R: TGCGTTACTGGGATAATGGG		1370	
	15	15F: ACTTAGCTTCGTCGCGTTAATGGC<br>15R: TCCTGTTCCGAATGAGCATGTTCAC		834	
	21	21F: TGTCTGTTAATGCTATCAACGG<br>21R: ATTGTTTATGCAGGGAATTTAGG		325	
Multiplex III	4	4F: TCATCTTTGCTGGAACACCAACGTA<br>4R: TGTGGGKATTGGAAATTACTTCGGG	60	311	[7]
	12	12F: GCTTGACGTACTCAAGGTTACGAGT<br>12R: TGATCAAGTTCGTTTAATTGAACCA		453	
	17	17F: ATACGGCTTTAGCAGGGCCA<br>17R: GTCGATGGGAGTTAACGCTG		640	
	19	19F: ATTCGTATTAGCCTCTGCAAATCCG<br>19R: TTCCAGCAAAATTTCCATCTTGGCG		796	
	23	23F: CCAATCTAGCATAGGATGGGAG<br>23R: ACATTCTTAGCCCAGGACCAGG		1082	
Multiplex IV	10/11	10/11F: TCCGCAAGGAAACCACCGTT<br>10/11R: GTTGCAGTAGCATTTCCAGTGTT	60	293	[7]
	16	16F: ACCATAGGTGATGCTTCAAAATCAGAACA<br>16R: TGGGTTGTTCAAACTCACAACACAA		1055	

Table S4. Primers used for detection of antimicrobial resistance genes in *E. rhusiopathiae*.

PCR type	Target gene	Primer sequence (5′→3′)	Amplicon size (bp)	Annealing temp. (°C)	Antimicrobial substances to which the gene confers resistance	Reference
multiplex	*aac(6')-Ie-aph(2")-Ia*	CAGAGCCTTGGGAAGATGAAG<br>CCTCGTGTAATTCATGTTCTGGC	348	56	GEN	[58-59]
	aph(3')-IIIa	GGCTAAAATGAGAATATCACCGG<br>CTTTAAAAAATCATACAGCTCGCG	523		KAN, NEO	
	ant(4')-Ia	CAAACTGCTAAATCGGTAGAAGCC<br>GGAAAGTTGACCAGACATTACGAA	294		NEO, KAN, TOB	
	aph(2")-Ib	CTTGGACGCTGAGATATATGAGCAC<br>GTTTGTAGCAATTCAGAAACACCCT	867		GEN	
	aph(2")-Ic	CCACAATGATAATGACTCAGTTCCC<br>CCACAGCTTCCGATAGCAAGAG	444		GEN	
	aph(2")-Id	GTGGTTTTTACAGGAATGCCATC<br>CCCTCTTCATACCAATCCATATAAC	641		GEN	
singleplex	*ant(6)-Ia*	CGGGAGAATGGGAGACTTTG<br>CTGTGGCTCCACAATCTGAT	563	56	STR	[60]
singleplex	*ant(9)-Ia*	GGTTCAGCAGTAAATGGTGGT<br>TGCCACATTCGAGCTAGGGTT	476	55	SPE	[60]
multiplex	*aadK*	GTCGCAATGAATGGTTCACGA<br>GCGATCAGTTGCCGGTATGA	348	54	STR	This study
	lsaE	TGTCAAATGGTGAGCAAACG<br>TGTAAAACGGCTTCCTGATG	496		TIA	[44]
	tetM	GTGGACAAAGGTACAACGAG<br>CGGTAAAGTTCGTCACACAC	406		TET	[61]
multiplex	*tetK*	GATCAATTGTAGCTTTAGGTGAAGG<br>TTTTGTTGATTTACCAGGTACCATT	155	60	TET	[61]
	tetL	TGGTGGAATGATAGCCCATT<br>CAGGAATGACAGCACGCTAA	229		TET	
	tetO	AACTTAGGCATTCTGGCTCAC<br>TCCCACTGTTCCATATCGTCA	515		TET	
	ermA	CCCGAAAAATACGCAAAATTTCAT<br>CCCTGTTTACCCATTTATAAACG	590		MACR, LIN	
	ermB	TGGTATTCCAAATGCGTAATG<br>CTGTGGTATGGCGGGTAAGT	745		MACR, LIN	
	mefA/E	CAATATGGGCAGGGCAAG<br>AAGCTGTTCCAATGCTACGC	317		MACR, LIN	
singleplex	*lnuB*	CCTACCTATTGTTTGTGGAA<br>ATAACGTTACTCTCCTATTC	925	50	LIN	[62]
singleplex	*Int-Tn* (Tn*916*/Tn*1545)*	GCGTGATTGTATCTCACT<br>GACGCTCCTGTTGCTTCT	1028	53	transposon	[51]
singleplex	*gyrA*	TCGTCTCCTATGCCATGTCG<br>AGTAAAAGTGCCCCTGTTGGA	613	54	ENR	This study

Legend: TET = tetracycline; MACR –macrolides; LIN – lincosamides; TIA – tiamulin; ENR – enrofloxacin; STR – streptomycin; SPE – spectomycin; GEN = gentamicin; KAN – kanamycin; NEO – neomycin;

Table S5. Bacterial strains used as positive controls for the detection of resistance genes.

Strain	Resistant gene	Accesion no. GenBank
Enterococcus faecalis 140	*tetO*	MK091469.1
Erysipelothrix rhusiopathiae 819	*lnuB*	MW428252
Lactobacillus salivarius 3aI	*tetL, tetM , ermB*	MK077521, MK077522, MK077523
Lactobacillus salivarius 5aI	*ant(6)-Ia*	MK091477
Lactobacillus salivarius 27eCh	*aph(2")-Ic*	MK091471
Lactobacillus salivarius Ch3a	*lsaE*	KY924692
Enterococcus faecium EC254	*mefA/E*	MN548247
Enterococcus faecalis 3W	*aph(3')-IIIa , ant(4')-Ia*<br>*aac(6')-Ie-aph(2")-Ia, int-Tn*	MK091474, MK091473,<br>MK091475, MK091481
Enterococcus faecium 60	*aph(2")-Id*	MK091472

Table S6. Primers used for detection of virulence-associated genes in *E. rhusiopathiae* strains.

Gene/ locus tag AP012027.1	Gene product	Primer sequence (5' – 3')	Position in genome of *E. rhusiopathiae* Fujisawa, Acc. No. AP012027.1	Annealing temp. [C]	Size [bp]	Reference
spaA, spaB, spaC	Surface protection antigen A	spaABC-F: ATGAAAAAGAAAAAACACCTATTTCCG	112931 to 112957		–	
spaA<br>ERH_0094	Surface protection antigen A	spaA-R: GCGATTTCTCCGCATAGCA	113725 to 113743	56 multiplex	813	[30]
spaB	Surface protection antigen B	spaB-R: CAATACCCTTTAGAGCCTCAACCA	Not applicable		904	
spaC	Surface protection antigen C	spaC-R: TGCCTCAACTACGGTTTGATACG	Not applicable		1011	
nanH.1/<br>ERH_0299	Neuraminidase	F: GGAGTGCCAGAAGGTACTTGG<br>R: TGAAGGATGCAGCGGATCAA	332530 to 332550<br>332768 to 332749	56	239	This study
nanH.2<br>ERH-0761	Neuraminidase	F: CGTATGGGTGCTGTTGCTTG<br>R: GCATGGCTTAGTGGCTACCT	803050 to 803069<br>803981 to 803962	55	932	This study
cpsA/<br>ERH_0157	Glycosyl transferase, capsule polysaccharide synthesis gene	F: ATGCGGATGTGCTTTTAGCG<br>R: GCAACTCTCCTTCACCTGCT	186420 to 186439<br>186685 to 186704	55	285	This study
ERH_1356	ABC transporter, metal-binding lipoprotein	F: ATGCTTGCAGGGTGTCAGT<br>R: TGCTTTGCAGTTGGCTCAC	1418969 to 1418987<br>1418332 to 1418350	53	656	This study
intI<br>ERH_1472	Internalin-like protein	F: ACAGTTTCGGATACTTCCGG<br>R: GCTGGTAAATATGACGAGGGT	1556207 to 1556226<br>1556515 to 1556535	53 multiplex	329	This study
rspA<br>ERH_0668	Rhusiopathiae surface protein A	F: ATCGACTGGTATTCAGTTGG R: ATCACGAGACATACCGCCAA	701593 to 701612<br>702108 to 702127		535	[16]
rspB<br>ERH_0669	Rhusiopathiae surface protein B	F: GTGGTTCATCACCACGTCCT<br>R: TGYAATTGTGAGTGCATCAGT	709112 to 709131<br>709582 to 709602	55	491	This study
algI<br>ERH_0402	Alginate-O-acetyltransferase	F: AGTTATCTTGGACTTGGTCC<br>R: AGATAAGTGCGTATTGATCC	432560 to 432579<br>431813 to 431832	50	767	This study
sub<br>ERH_0260	Cell-envelope associated proteinase, subtilase family	F: AAGCCTGAGATATCTGCACC<br>R: TTGTACAATTGGATGAGCCG	297956 to 297975<br>298164 to 298183	53	226	[16]
hlyA<br>ERH_0150	Hyaluronidase	F: AGGATCACTTACCGCTATGG<br>R: CAGCACTCAGCATGTTCTC	176491 to 176510<br>177413 to 177431		941	[16]
fbpA<br>ERH_1034	Fibronectin-binding protein	F: ATCTCGCCGCTTTTAGAACG<br>R: GCGTCTTCAACTGTTGCTTG	1092921 to 1092940<br>1093503 to 1093522	53 multiplex	602	[16]
hlyIII<br>ERH_0649	Hemolysin	F: TACGATTGCGACAAAGTGTGCG<br>R: CAGCCTTCCCTATGTTTCCAT	678297 to 678318<br>678820 to 678840		544	This study

Table S7. List of *E. rhusiopathiae* strains whose *spaA* gene sequences were used for comparative analysis and clustering.

No.	Strain ID	Source	Serotype	Year of isolation	Country of isolation	GenBank Acc. No.	Reference
1	NCTC 8163 (ATCC 19414)	pig	2	1950	United Kingdom	LR134439.1	Unpublished data
2	R32E11	unknown	2	<1970*	Unknown	MZ448116	This study
3	Ireland	pig	6	ND	Japan	LC425606.1	[33]
4	AQ 150414	pig	1a	ND	China	KU214208.1	Unpublished data
5	422/1E1	pig	1b	ND	Japan	AB259653.1	[17]
6	C43065	pig	2	ND	China	EF688017.1	[63]
7	Fujisawa	pig	1a	<1972	Japan	AP012027.1	[9]
8	ZJ	pig	ND	ND	China	CP041995.1	[48]
9	GD-GZ	pig	1a	ND	China	KJ660060.1	Unpublished data
10	Pecs 67	pig	5	<1992	Japan	AB259655.1	[17]
11	759W	goose	8	2019	Poland	MZ448126	Unpublished data
12	1092	goose	8	2021	Poland	OM248659	Unpublished data
13	1023	goose	2	2021	Poland	OP822697	Unpublished data
14	48W	goose	5	2019	Poland	MZ448141	Unpublished data
15	219	goose	2	2020	Poland	MZ448138	Unpublished data
16	849	goose	6	2020	Poland	MZ448135	Unpublished data
17	49W	goose	N	2019	Poland	MZ448148	Unpublished data
18	1S	pig	N	2017	Poland	OP822679	This study
19	2S	pig	6	2018	Poland	OP822680	This study
20	3S	pig	1b	2018	Poland	OP822681	This study
21	4S	pig	1b	2019	Poland	OP822682	This study
22	5S	pig	2	2019	Poland	OP822683	This study
23	6S	pig	5	2019	Poland	OP822684	This study
24	7S	pig	1b	2019	Poland	OP822685	This study
25	8S	pig	8	2019	Poland	OP822686	This study
26	9S	pig	2	2019	Poland	OP822687	This study
27	10S	pig	5	2018	Poland	OP822688	This study
28	11S	pig	1b	2017	Poland	OP822689	This study
29	12S	pig	1b	2018	Poland	OP822690	This study
30	13S	pig	1b	2018	Poland	OP822691	This study
31	14S	pig	2	2022	Poland	OQ054982	This study

* The country and period of isolation of the strain was determined on the basis of information ND – not determined
